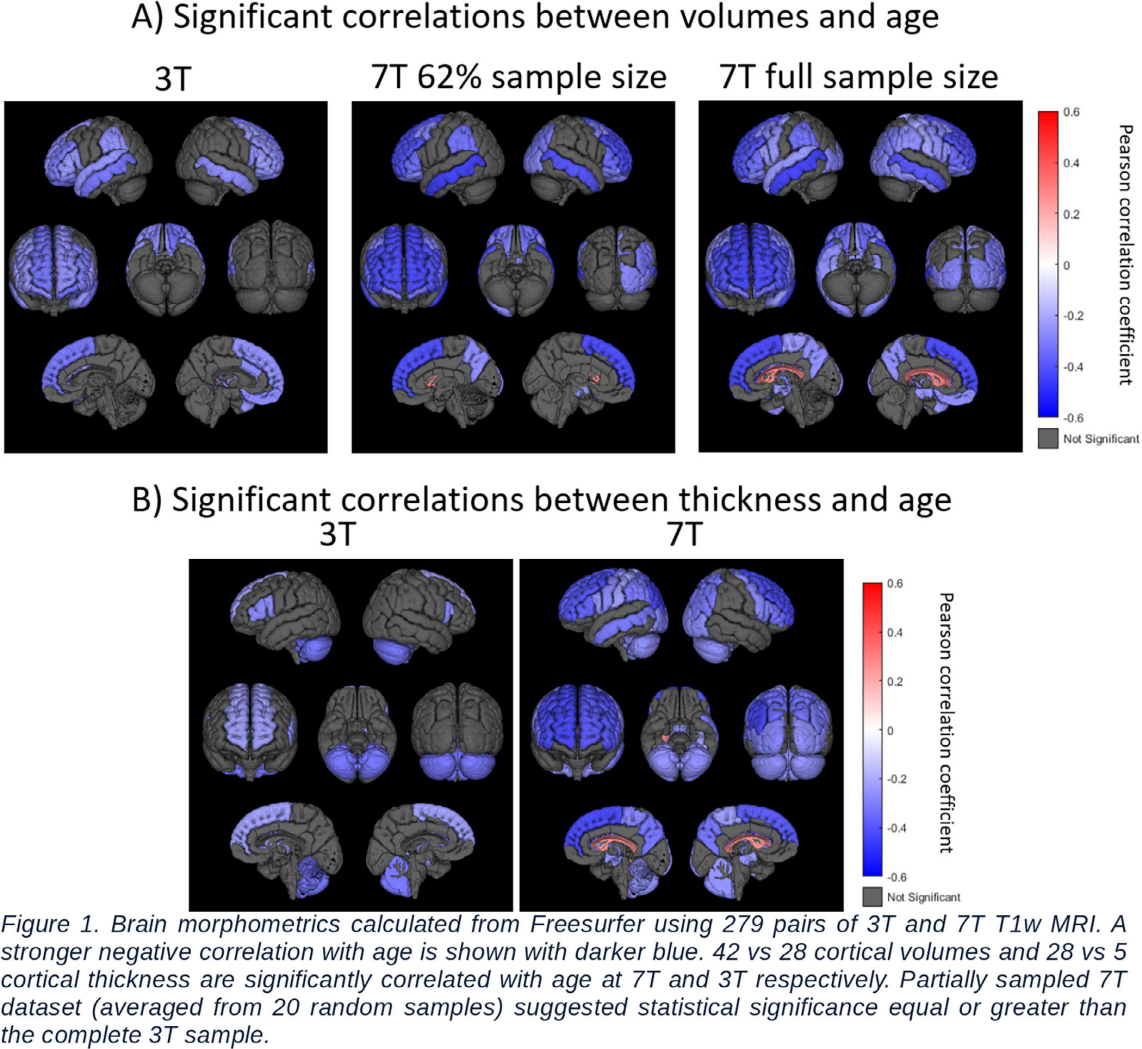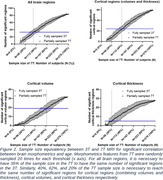# Brain morphometrics and aging with paired 3T and 7T structural MRI: 7T reduces reqiured sample size and improves statistical significance

**DOI:** 10.1002/alz.093423

**Published:** 2025-01-09

**Authors:** Cong Chu, Tales Santini, Anna Marsland, Peter Gianaros, Tamer S Ibrahim

**Affiliations:** ^1^ University of Pittsburgh, Pittsburgh, PA USA

## Abstract

**Background:**

During normal aging, it has been observed that brain regional volume and thickness can be altered due to atrophy. While both 3 Tesla (3T) and 7 Tesla (7T) MRI are used to study such changes, it is unknown how individual brain morphology is characterized differently when 7T MRI allows for higher‐resolution and higher tissue contrast images. In this study, we employ a large cohort of healthy subjects that have undergone both 3T and 7T MRI scans to investigate how 7T MRI improves correlation strength between brain morphometrics and aging.

**Method:**

Paired 3T (Prisma and Trio Siemens) and 7T (TTT coil [Santini et al Sci. Rep. 2021] with Magnetom Siemens) images (T1‐weighted, 0.5x0.5x1mm^3^, 1x1x1mm^3^, 0.75x0.75x0.75mm^3^ respectively) are acquired with similar acquisition time (∼5 minutes) on 351 healthy participants (age=46.7 +‐ 9.12 years, 192 females). Images are preprocessed with gradient distortion correction, bias correction, and skull stripping. 279 pairs of images passed manual quality assurance without abnormalities. We used Pearson correlation coefficient to assess associations between brain morphometrics and age. Regional volumes are normalized to individual intracranial volume. Multiple tests were corrected using the Bonferroni method.

**Result:**

Compared to 3T, the 7T dataset showed more significantly correlated regions, with 28 volume regions in common to 3T (average R_3T_=‐0.24 and R_7T_=‐0.38) and 14 more. 7T dataset also consistently exhibits stronger negative and positive (ventricles) correlation with age than 3T for both volume and thickness. The 62% subsampled 7T dataset, averaged from 20 random samples, resembled the significant volume regions of the 3T dataset while still showing stronger correlation (Figure 1). We also show that for all brain regions, volume and thickness, 35% of the 7T sample is sufficient to reach the statistical power of the complete 3T sample (Figure 2).

**Conclusion:**

Our analysis demonstrated the advantages of 7T MRI at characterizing brain morphometrics, specifically its capacity to measure relatively small changes without needing a large sample size. The higher spatial resolution and signal to noise ratio allow for feasible longitudinal, more importantly with relatively short follow up times, and cross‐sectional analysis with reasonable sample sizes.